# Challenges and potential of PD-1/PD-L1 checkpoint blockade immunotherapy for glioblastoma

**DOI:** 10.1186/s13046-019-1085-3

**Published:** 2019-02-18

**Authors:** Xin Wang, Gaochao Guo, Hui Guan, Yang Yu, Jie Lu, Jinming Yu

**Affiliations:** 1 Department of Oncology, Renmin Hospital of Wuhan University, Wuhan, 430060 Hubei Province China; 2 Department of Radiation Oncology, Shandong Cancer Hospital Affiliated to Shandong University, Shandong Academy of Medical Sciences, Jinan, 250117 Shandong Province China; 3 Department of Neurosurgery, Tianjin Medical University General Hospital, Tianjin, China; 4 Key Laboratory of Post-Trauma Neuro-Repair and Regeneration in Central Nervous System, Ministry of Education, Tianjin, China; 5 Tianjin Key Laboratory of Injuries, Variations and Regeneration of Nervous System, Tianjin, China; 6 Department of Radiation Oncology, The Fourth People’s Hospital of Jinan, Jinan, Shandong Province China; 7 Department of Neurosurgery, Shandong Province Qianfoshan Hospital of Shandong University, Shandong Province, Jinan, 250014 China

**Keywords:** Glioblastoma multiforme, Nivolumab, Tumour infiltrating lymphocytes, Tumour mutation load, Temozolomide

## Abstract

PD-1/PD-L1 checkpoint blockades have achieved significant progress in several kinds of tumours. Pembrolizumab, which targets PD-1, has been approved as a first-line treatment for advanced non-small cell lung cancer (NSCLC) patients with positive PD-L1 expression. However, PD-1/PD-L1 checkpoint blockades have not achieved breakthroughs in treating glioblastoma because glioblastoma has a low immunogenic response and an immunosuppressive microenvironment caused by the precise crosstalk between cytokines and immune cells. A phase III clinical trial, Checkmate 143, reported that nivolumab, which targets PD-1, did not demonstrate survival benefits compared with bavacizumab in recurrent glioblastoma patients. Thus, the combination of a PD-1/PD-L1 checkpoint blockade with RT, TMZ, antibodies targeting other inhibitory or stimulatory molecules, targeted therapy, and vaccines may be an appealing solution aimed at achieving optimal clinical benefit. There are many ongoing clinical trials exploring the efficacy of various approaches based on PD-1/PD-L1 checkpoint blockades in primary or recurrent glioblastoma patients. Many challenges need to be overcome, including the identification of discrepancies between different genomic subtypes in their response to PD-1/PD-L1 checkpoint blockades, the selection of PD-1/PD-L1 checkpoint blockades for primary versus recurrent glioblastoma, and the identification of the optimal combination and sequence of combination therapy. In this review, we describe the immunosuppressive molecular characteristics of the tumour microenvironment (TME), candidate biomarkers of PD-1/PD-L1 checkpoint blockades, ongoing clinical trials and challenges of PD-1/PD-L1 checkpoint blockades in glioblastoma.

## Introduction

Glioblastoma is the most common and malignant brain tumour due to its aggressive biological behaviour and resistance to treatment. Glioblastoma has a morbidity rate of 0.59–3.69/100000 people worldwide, with a median onset of 63.0 years. The age-adjusted morbidity is 3.97/100000 for males and 2.53/100000 for females [1–3]. The standard therapies according to NCCN Guidelines include tumour resection, radiotherapy with concomitant temozolomide (TMZ) and adjuvant TMZ chemotherapy, with the combination of radiotherapy and other therapies, the 5-year overall survival was 9.8% versus 1.9% with radiotherapy alone. Although with standard therapy, the median survival time of GBM patients is only 12–15 months after diagnosis [4, 5].

With growing evidence supporting the dynamic interaction between the central nervous system (CNS) and the systemic immune system, the conventional doctrine proclaiming immunoprivilege of the CNS has been abandoned [6, 7]. Considering that PD-1/PD-L1 checkpoint blockades have dramatically changed the treatment patterns for advanced non-small cell lung cancer (NSCLC), renal cancer, chronic Hodgkin’s lymphoma, gastric cancer, urothelial cancer, cervical cancer, head and neck squamous cell carcinoma, hepatocellular carcinoma, and melanoma [8–12], more explorations of immune checkpoint inhibitors in glioblastoma have been conducted. Several studies have shown that PD-L1 is highly expressed on glioblastoma cells [13, 14], and combinational checkpoint blockade immunotherapy has demonstrated promising efficacy in preclinical glioblastoma mouse models [15–17]. However, the clinical efficacy of the PD-1/PD-L1 checkpoint blockade in glioblastoma is controversial. All of these studies demonstrated that the PD-1/PD-L1 pathway plays only a role in the malignant biological behaviour of glioblastoma but that other molecular signalling networks may also play indispensable roles. Thus, exploring effective targets in the TME and combination therapies to improve the clinical response of PD-1/PD-L1 checkpoint blockades is urgently needed.

### PD-L1 expression and clinical efficacy of PD-1/PD-L1 checkpoint blockades in glioblastoma

The PD-1/PD-L1 pathway plays an important role in suppressing the function of T cells in eradicating tumour cells [18–20]. PD-L1 is upregulated in several types of solid tumours, and high expression levels of PD-L1 often indicate better clinical efficacy of PD-1/PD-L1 checkpoint blockades [21–24]. Although this phenomenon is not ubiquitous, it still drives us to explore the connection between PD-L1 expression and the clinical efficacy of PD-1/PD-L1 checkpoint blockades in glioblastoma patients.

Berghoff et al. observed PD-L1 expression in 88% of newly diagnosed and 72.2% of recurrent glioblastoma specimens [13]. Similarly, Nduom et al. measured PD-L1 expression in 94 glioblastoma patients and found that 61% of patients had tumours with PD-L1 positive cells [14]. A phase I cohort of Checkmate 143 evaluated the safety, tolerability and clinical effects of nivolumab with or without ipilimumab (which targets CTLA-4) in recurrent glioblastoma patients. Among all 40 patients, 10 patients were randomized to receive nivolumab 3 mg/kg every 2 weeks, 10 patients received nivolumab 1 mg/kg + ipilimumab 3 mg/kg every 3 weeks for 4 doses, and the other 20 patients received nivolumab 3 mg/kg + ipilimumab 1 mg/kg every 3 weeks for 4 doses. Omuro et al. demonstrated that the subgroup receiving nivolumab 3 mg/kg tolerated the treatment better than other subgroups that received the other combinations of nivolumab 1 mg/kg + ipilimumab 3 mg/kg and nivolumab 3 mg/kg + ipilimumab 1 mg/kg (90% vs 70% vs 80%). Other than fatigue and diarrhoea, which were the most common treatment-related adverse events (AEs) (30% vs 80% vs 55%; and 10% vs 70% vs 30%, respectively), no other side effects were observed. Nivolumab monotherapy was better tolerated than combination therapy. Simultaneously, we found that the dose of the ipilimumab monoclonal antibody was negatively correlated with patient tolerance, which may be explained by the critical role of the ipilimumab antibody in the earlier phase of T cell activation that can cause an extensive impact in the immune network [25]. The phase III clinical trial Checkmate 143 reported that PD-1 monoclonal antibody (nivolumab) monotherapy does not improve overall survival (OS) time compared with bavacizumab therapy in recurrent glioblastoma patients who were previously treated with chemotherapy and radiotherapy. The median PFS was 1.5 months for nivolumab vs 3.5 months for bavacizumab, the median OS was 9.8 months for nivolumab vs 10.0 months for bavacizumab, and the objective response rate (ORR) was 8% months for nivolumab vs 23% months for bavacizumab [26]. One possible reason for the failure of nivolumab monotherapy may be lymphopenia caused by radiotherapy. Yovino et al. found that after RT with 30 conventional fractions of 2 Gy, the circulating lymphocytes received a 2.2 Gy mean dose, and 99% of the circulating lymphocytes received mean doses ≥0.5 Gy [27]. However, nivolumab monotherapy exerts an immune activation effect through competitive binding with the PD-1 receptor on lymphocytes. Another possible reason may be the anergic nature of effector T cells to tumour-specific antigens in the TME. Furthermore, Wherry et al. examined the phenotypes of tumour infiltrating lymphocytes (TILs) in glioma specimens and found phenotypes rich in CD95, PD-1, PD-L1, CTLA-4, LAG3, and TIM-3, which obviously indicated the immune exhaustion of T cells [28]. In addition, Reardon et al. [17] also found that TILs express immunoinhibitory molecules, including CTLA-4 and PD-1, or coexpress PD-1 and TIM-3. However, the PD-1+/TIM-3+ phenotype represents an exhausted CD8+ T cell population in tumours [29]. Considering the low immunogenic characteristics and complicated immunosuppressive networks in glioblastoma, PD-1 checkpoint blockades are unlikely to overcome the factors leading to T cell anergy.

Based on the phase III clinical trial results, Checkmate 143 reported that nivolumab did not exhibit increased survival benefits over bavacizumab, researchers then explored the clinical efficacy of nivolumab + RT ± TMZ in newly diagnosed glioblastoma patients in ongoing phase III clinical trials, including Checkmate 498 (NCT02617589) and Checkmate 548 (NCT02667587). Checkmate 498 is comparing the efficacy of nivolumab + RT versus the standard treatment of TMZ + RT in newly diagnosed glioblastoma patients with unmethylated MGMT. Checkmate 548 is comparing the efficacy of nivolumab + RT + TMZ versus RT + TMZ in newly diagnosed glioblastoma patients with methylated MGMT. The clinical trials on glioblastoma are summarized in Table 1.

**Table 1:** Clinical trials of PD-1/PD-L1 checkpoint blockades in glioblastoma

Setting	Trials	No.	Arms	Characteristic	Target	Phase	Results
Neoadjuvant glioblastoma	NCT 02550249	29	Nivo+continued surgery	Primary and recurrent glioblastoma	PD-1	II	–
	NCT 02852655	35	Pem + surgery+Pem	Recurrent/Progressive glioblastoma	PD-1	NA	–
	NCT 02337686	18	Pem + surgery+Pem	Recurrent glioblastoma	PD-1	II	–
Newly diagnosed glioblastoma	NCT 02667587	550	Nivo+RT vs TMZ + RT	Unmethylated MGMT	PD-1	III	–
	NCT 02617589	693	Nivo+RT + TMZ vs RT + TMZ	MGMT-methylated	PD-1	II	–
	NCT 03047473	30	Ave + RT + TMZ		PD-L1	II	–
	NCT 02530502	50	Pem + RT + TMZ		PD-1	I/II	–
	NCT 02311920	32	Arm I: TMZ + Ipi; Arm II: TMZ + Nivo; Arm III: TMZ + Nivo+Ipi		PD-1/CTLA-4	I	–
	NCT 03347097	40	PD-1-PIK T cells		PD-1	I	–
Recurrent glioblastoma	NCT 02658279	44	Pem	Hypermutator phenotype	PD-1	NA	–
	NCT 02968940	43	Ave + HFRT	IDH mutant glioblastoma		II	–
	NCT 02311582	58	Pem + MLA vs Pem		PD-1	I	–
	NCT 02430363	58	Pem vs inhibitors of PI3K/Akt pathway		PD-1	I/II	–
	NCT02054806	26	Pem	PD-L1 expression≥1%	PD-1	I	mPFS:2.8 m; mOS:14.4 m; G3–4 TRAEs:15.4%
	NCT 02336165	159	Arm A: MEDI4736 + RT; Arm B: MEDI4736; Arm B2: MEDI4736 + Bev (10 mg/Kg); Arm B3: MEDI4736 + Bev (3 mg/Kg); Arm C: MEDI4736 + Bev	Arm A: unmethylated MGMT Arm B: Bev-naïve Arm B2: Bev-naïve Arm B3: Bev-naïve Arm C: Bev-refractory	PD-L1/VEGF	II	ArmB:6 m-PFS:20%; 12 m-OS:44.4%; G3–4 TRAEs:9.7%; ArmC: OS≥22 week: 36%; PFS ≥ 8 weeks: 50%; G3–4 TRAEs: 4.5%
	NCT 02337491	80	Pem + Bev vs Pem		PD-1/VEGF	II	Safety; mOS: 6.8 m
	NCT 02794883	36	Dur vs Tre + Dur		PD-L1/CTLA-4	II	–
	NCT 02017717	369 40	Nivo vs Bev Nivo vs Nivo+Ipi (Nivo3mg = 10; Nivo1mg + Ipi3mg = 10; Nivo3mg + Ipi1mg = 20)		PD-1/CTLA-4/VEGF	III I	mPFS: 1.5 m vs 3.5 m; mOS: 9.8 m vs 10.0 m; ORR: 8% vs 23%; G3–4 TRAEs 18% vs 15% Safety; Nivo3mg better Tolerated than other combinations 12 m-OS: Nivo3mg: 40%; Nivo1mg + Ipi3mg: 30%; Nivo3mg + Ipi1mg: 35%
	NCT 02658981	100	Arm A1: Anti-LAG-3; Arm A2: Anti-CD137; Arm B1: Anti-LAG3 + Nivo; Arm B2: Anti-CD137 + Nivo		PD-1/LAG-3/CD137	I	–
	NCT 02335918	175	Var + Nivo		PD-1/CD27	II	–
	NCT 02937844	20	Anti-PD-L1 CSR T cells		PD-L1	I	–
OVT	NCT 02798406	48	DNX-2401+ Pem	Recurrent glioblastoma and GS	PD-1	II	–
Radiotherapy	NCT 02648633	4	Valproate+SRS + Nivo	Recurrent glioblastoma	PD-1	I	–
	NCT 02313272	23	HFSRT+Pem + bev	High grade gliomas (III and IV)	PD-1/VEGF	I	6 m-OS:94%;12 m-OS:64%; ORR:53%
	NCT 02829931	26	HFSRT+Ipi + Nivo+Bev	Recurrent high grade gliomas	PD-1/CTLA-4/VEGF	I	–
	NCT 02866747	62	HFSRT vs HFSRT+Dur	Recurrent glioblastoma	PD-L1	I/II	–
		20	Pem/Nivo+RT	Recurrent high grade gliomas	PD-1		mPFS:4 m; mOS:10 m; ORR:35%;
Tumor vaccines	NCT 02529072	7	Arm A: Nivo+surgery+Nivo and DC vaccine; Arm B: Nivo and DC vaccine+surgery + Nivo and DC vaccine	Recurrent high grade gliomas	PD-1	I	–
	NCT 03422094	30	NeoVax+Nivo/NeoVax+Nivo+Ipi	Newly diagnosed glioblastoma	PD-1	I	–
	NCT 03018288	108	RT + TMZ + Pem + HSPPC-96 vs RT + TMZ + Pem	Newly diagnosed glioblastoma	PD-1	II	–
	NCT 03014804	30	DCVax-L vs DCVax-L + Nivo	Recurrent glioblastoma	PD-1	II	–
anti-CSF-1R	NCT 02526017	295	Cabiralizumab+Nivo	glioblastoma	PD-1	I	–

*GS* Gliosarcoma, *Nivo* Nivolumab, *Anti-PD-1* Antibody, *Pem* Pembrolizumab, *Anti*-*PD-1* Antibody, *TMZ* Temozolomide, *Ave* Avelumab, *Anti-PD-L1* Antibody, *PD-1*-*PIK T cells* Pluripotent immune killer T cells express PD-1 antibody, *HFRT* Hypofractionated radiation therapy, *IDH* Isocitrate Dehydrogenase, *MLA* MRI-guided laser ablation, *Ipi* Ipilimumab, *Anti-CTLA-4* Antibody, *VEGF* Vascular endothelial growth factor, *Tre* Tremelimumab, *Anti-CTLA-4* Antibody, *Dur* Durvalumab, *Anti-PD-L1* Antibody, *Var* Varlilumab, *Anti-CD27* Antibody, *OVT* Oncolytic virotherapy, *HFSRT* Hypofractionated stereotactic irradiation, *Anti-PD-L1 CSR T cells* Autologous Chimeric Switch Receptor Engineered T Cells Redirected to PD-L1, *DNX-2401* A genetically modified oncolytic adenovirus, *DC* Dendritic cell, *HSPPC-96* a vaccine made from fresh tumor taken at the time of surgery, *DCVax-L* Autologous DC pulsed with tumor lysate antigen Vaccine, *Cabiralizumab* Anti-CSF-1R antibody

### Cellular and molecular characteristics of the microenvironment in glioblastoma

**Fig. 1:**
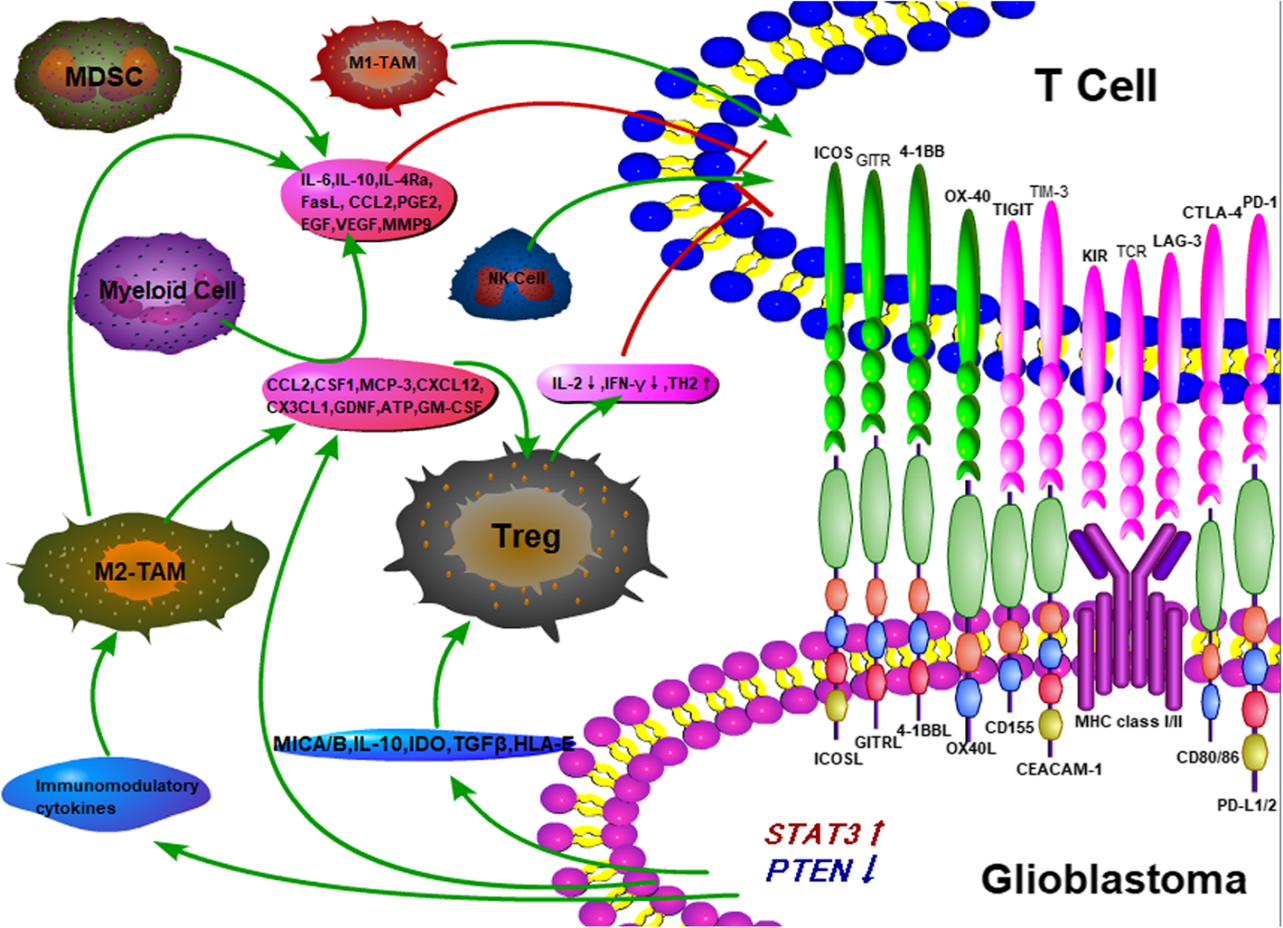
The immunosuppressive mechanism of glioblastoma microenvironment. The immunosuppressive microenvironment of glioblastoma is composed of a variety of immunosuppressive cells and cytokines. The effective immune cells mainly include CD4+ T cells, CD8+ T cells, NK cells, and tumour-inhibiting M1-TAMs, which are in a state of exhaustion or suppression in the microenvironment. The immunosuppressive cells mainly include Tregs, tumourigenic M2-TAMs, myeloid cells, and MDSCs. Tumour cells express high levels of PD-L1 and IDO, downregulate MHC and costimulatory molecules, express/activate STAT3, cause PTEN loss, then reduce the immunogenicity and induce recruitment of Tregs. Tumour cells secrete MICA/B, IL-10, TGF-β, and HLA-E to recruit Tregs and inhibit both T cell and NK cell activity. Through the secretion of diverse chemokines and other factors, such as CCL2, CSF1, MCP-3, CXCL12, CX3CL1, GDNF, ATP, and GM-CSF, the paracrine network signalling between glioblastoma and the TAMs attracts myeloid cells and infiltrates Tregs. Furthermore, tumour cells secrete immunomodulatory cytokines that polarize TAMs to the immunosuppressive M2 phenotype. Immunosuppressive cells, including M2-TAMs, myeloid cells, and MDSCs, secrete a variety of cytokines (IL-6, IL-10, IL-4Ra, FasL, CCL2, PGE2, EGF, VEGF, and MMP9) to suppress the function of cytotoxic T lymphocytes (CTLs) and promote the progression of tumour cells. In addition, Tregs downregulate IL-2 production, inhibit IFN-γ production, and upregulate T_H_2 cytokine secretion to inhibit T cell function. TAM: tumor-associated macrophage; MDSC: myeloid-derived suppressor cell; CCL2: chemokine ligand 2; CSF1: colony-stimulating factor 1; MCP-3: monocyte-chemotactic protein-3; GDNF: glial cell-derived neurotrophic factor; GM-CSF: granulocyte-macrophage colony-stumulating factor; KIR: killer cell Ig-like receptor; GITR: glucocorticoid-induced TNFR-related protein; STAT3: signal transducers and activators of transcription; PGE2: prostaglandin E2; EGF: epidermal growth factor; VEGF: vascular endothelial growth factor; MMP9: matrix metalloproteinase-9

Glioblastoma is highly heterogeneous with intratumoural heterogeneity and intertumoural heterogeneity. According to the 2016 CNS WHO classification, glioblastomas are divided into glioblastoma, IDH-wild type and glioblastoma, IDH-mutant type based on molecular pathology [30]. Approximately 90% of glioblastomas are IDH-wild type, which indicates a worse prognosis, and approximately 10% of glioblastomas are IDH-mutant type, which indicates a better prognosis [31]. In addition, glioblastoma has been divided into four major subtypes based on genomic discrepancies: (1) neural, (2) pro-neural (PN), (3) classical (CL), and (4) mesenchymal (MES) [32]. These four subtypes have distinct cellular and molecular characteristics in their respective microenvironments. For example, NF1 and TP53 deletions and mutations were found in classical type, PDGFRA amplification and IDH1 point mutation were found in pro-neuronal type and EGFR overexpression was found in neuronal type [32]. Thus, finding therapeutically targetable genes that are expressed by all four subtypes is challenging. For example, Wang et al. analysed immune cell types in human PN, CL, and MES samples and found that CD4+ memory T cells, type-2 polarized macrophages (M2), and neutrophils were commonly increased in the MES subtype but not in the other subtypes [33]. Furthermore, Berghoff et al. demonstrated that the MES subtype of glioblastoma has higher PD-L1 expression [13]. Despite the genomic discrepancies and distinct cellular and molecular characteristics in the four subtypes, glioblastoma ubiquitously exhibited an immunosuppressive microenvironment that involves a number of tumour-cell-intrinsic and tumour-cell-extrinsic factors [34]. In contrast to NSCLC and melanoma, which have higher levels of tumour mutational load (TML) [35, 36], glioblastoma exhibits a lower TML in most instances and infrequently shows a high TML when it is deficient in MMR protein and there is an exonuclease proof-reading domain of the DNA polymerase epsilon gene (POLE) mutation. Thus, varying sensitivities to PD-1/PD-L1 checkpoint blockades may also be observed in glioblastoma. Furthermore, neoantigens represent tumour-specific mutant antigens encoded by somatic mutations in the cancer genome. The low neoantigen burden in glioblastoma reduced the chances of the immune system overcoming central tolerance to recognize tumour cells [37]. In addition, some specific gene mutations in glioblastoma induced an immunosuppressive microenvironment through regulating the crosstalk between cytokines and immune cells [14, 33, 38–46]. The immunosuppressive microenvironment of glioblastoma is composed of a variety of immunosuppressive cells and cytokines. The effective immune cells mainly include CD4+ T cells, CD8+ T cells, NK cells, and tumour-inhibiting M1-TAMs, which are in a state of exhaustion or suppression in the microenvironment. The immunosuppressive cells mainly include Tregs, tumourigenic M2-TAMs, myeloid cells, and MDSCs. Tumour cells express high levels of PD-L1 and IDO, downregulate MHC and costimulatory molecules, express/activate STAT3, cause PTEN loss, then reduce the immunogenicity and induce recruitment of Tregs. Tumour cells secrete MICA/B, IL-10, TGF-β, and HLA-E to recruit Tregs and inhibit both T cell and NK cell activity. Through the secretion of diverse chemokines and other factors, such as CCL2, CSF1, MCP-3, CXCL12, CX3CL1, GDNF, ATP, and GM-CSF, the paracrine network signalling between glioblastoma and the TAMs attracts myeloid cells and infiltrates Tregs. Furthermore, tumour cells secrete immunomodulatory cytokines that polarize TAMs to the immunosuppressive M2 phenotype. Immunosuppressive cells, including M2-TAMs, myeloid cells, and MDSCs, secrete a variety of cytokines (IL-6, IL-10, IL-4Ra, FasL, CCL2, PGE2, EGF, VEGF, and MMP9) to suppress the function of cytotoxic T lymphocytes (CTLs) and promote the progression of tumour cells. In addition, Tregs downregulate IL-2 production, inhibit IFN-γ production, and upregulate T_H_2 cytokine secretion to inhibit T cell function [34, 47–51]. The molecular characteristics of the TME in glioblastoma patients are depicted in Fig. 1. Thus, the exploration of combination therapy based on PD-1/PD-L1 checkpoint blockades is important to the study of glioblastoma.

### Candidate choice for combination therapy

T cells in the TME normally exhibit an exhausted phenotype with reduced effector function. Negative regulatory signals resulting from the activation of multiple inhibitory checkpoints that block T cells are the primary mechanism leading to effector T cell dysfunction [52]. Studies have shown that inhibitory checkpoints could reverse the exhausted phenotype of effector T cells [53, 54]. Although PD-1 receptor expression is an important factor for the degree of T cell exhaustion, many patients with tumours are still unable to benefit from PD-1/PD-L1 checkpoint blockades. Exhausted T cells in the TME typically express multiple checkpoints, and blockade of a single checkpoint is not sufficient to activate the suppressed immune response [55], this is especially true in glioblastoma, which has a higher degree of immunosuppression in the TME. Therefore, it is urgent to explore a combination treatment regimen of checkpoint blockades and other regimens with a higher response rate. Since PD-1 checkpoint blockades do not significantly benefit patients with relapsed glioblastoma in OS compared with bevacizumab, exploring candidate targets related to the immune response may provide new strategies associated with promoting the clinical efficacy of PD-1/PD-L1 checkpoint blockades.

#### Cytotoxic T lymphocyte-associated antigen-4 (CTLA-4)

The expression of CTLA-4 increased significantly after T cell activation, and CTLA-4 reduced the early stages of T cell expansion by competing to bind to B7 ligands in systemic lymph nodes [56, 57]. Reardon et al. demonstrated that combinatorial therapy targeting CTLA-4 and PD-1 could cure 75% of mice, including those with advanced-stage tumours, and induce tumour-specific memory effects to overcome tumour recurrences. The cure rates of blockades against PD-1, PD-L1, or CTLA-4 alone were 50, 20, and 15%, respectively. This combination strategy significantly increased activated CD8+ T cells and NK cells and decreased suppressive CD4 + FoxP3 + Treg cells both in the TME and in draining lymph nodes [17]. This result suggests that the combined PD-1 and CTLA-4 checkpoint blockades could relieve the inhibition of T cell function during the activation phase and effect phase. However, Checkmate 143 reported that PD-1 checkpoint blockade (nivolumab) in combination with CTLA-4 checkpoint blockade (ipilimumab) resulted in 40% of relapsed glioblastoma patients having intolerable treatment-related severe side effects. The toxic side effects limit the widespread use of nivolumab therapy in combination with ipilimumab therapy. Thus, a phase I study (NCT03527251) aimed to evaluate the safety and efficacy of the CTLA-4 antibody followed by the PD-1 antibody in patients with recurrent or metastatic non-small cell lung cancer. The same treatment strategy is also worth exploring in GBM.

#### TIM-3 (T cell immunoglobulin domain and mucin domain-3)

TIM-3 is an inhibitory receptor and a surface protein that is selectively expressed on CD4 + T-helper 1 and CD8+ T cytotoxic cells that causes T cell failure in tumour progression and chronic virus infection [58–60]. In immunocompetent mouse models, Reardon et al. [17] found that TILs express immunoinhibitory phenotypes, including CTLA-4, PD-1 or the coexpression of PD-1 and TIM-3. However, the PD-1+/TIM3+ phenotype represents an exhausted CD8+ T cell population in tumours [29]. Exhausted CD8+ T cells exhibit poor effector function and became anergic to specific tumour antigen stimulation. Simultaneously, the resistance to PD-1 checkpoint blockades was prevented when an anti-TIM-3 antibody was added to the treatment. A multicentre phase I study (NCT02817633) evaluating the anti-TIM-3 antibody TSR-022 combined with an anti-PD-1 antibody is recruiting patients with advanced solid tumours who have limited available treatment options. Furthermore, a phase II study (NCT03680508) is studying how well TSR-022 (anti-TIM-3 antibody) and TSR-042 (anti-PD-1 antibody) work in combination in treating patients with locally advanced or metastatic liver cancer. Thus, combination therapy targeting PD-1 and TIM-3 may be a potential strategy to overcome T cell anergy.

#### LAG-3 (lymphocyte activation gene-3)

Similar to PD-1, LAG-3 is also an inhibitory receptor that is expressed on the surface of T cells, B cells, nature killer (NK) cells, and dendritic cells (DC). LAG-3 downregulates T cell activity by binding to the main histocompatibility complex class II (MHC class II) [61]. In addition, LAG-3 also enhances the intrinsic inhibitory activity of Tregs. LAG-3 is another important tumour immune checkpoint that may have synergistic effects with the PD-1/PD-L1 pathway [62]. A phase I study (NCT03250832) evaluated the anti-LAG-3 antibody TSR-033 alone and in combination with an anti-PD-1 antibody.

#### IDO (Indoleamine 2,3 dioxygenase)

IDO is an intracellular enzyme that plays an immunosuppressive role, inhibits T cell proliferation and causes T cell apoptosis and Treg accumulation by reducing tryptophan levels [63, 64]. Sordillo et al. demonstrated IDO overexpression in glioblastoma specimens, and IDO upregulation was significantly associated with a poor prognosis [65]. Furthermore, Wang et al. observed IFN-γ-induced IDO upregulation [66]. IDO was responsible for mediating the adaptive resistance of tumours to PD-1/PD-L1 or CTLA-4 checkpoint blockades [67]. Thus, targeting IDO may be a potential strategy to augment the clinical efficacy of PD-1/PD-L1 checkpoint blockades. A phase I study (NCT03491631) was designed to characterize the effect of PD-1 checkpoint blockades in combination with IDO inhibitors in patients with advanced solid tumours. Another phase I study (NCT03343613) evaluated the safety of IDO inhibitors alone or in combination with PD-L1 checkpoint blockades in patients with solid tumours.

#### 4-1BB (CD137)

4-1BB, a co-stimulatory receptor expressed on both T cells and antigen presenting cells, could augment cytotoxic CD8 T cells and modulate the activity of CD4 T cells, B cells, NK cells, monocytes, and antigen presenting cells to potentiate the antitumour immunity of T cells [68]. Shindo et al. explored the efficacy of a 4-1BB agonist antibody in combination with PD-1 checkpoint blockade compared with a single agent in mouse models with CT26 tumour cells and found that the combination therapy had the best antitumour response that resulted in complete tumour rejection [69]. A phase Ib study (NCT02179918) evaluated the efficacy of the 4-1BB agonist utomilumab in combination with the PD-1 checkpoint blockade pembrolizumab in patients with advanced solid tumours. Among all 23 patients, six (26.1%) patients were responders, and none of the patients who received combination treatment showed dose-limiting toxicities [70]. This study demonstrated that this combination strategy may be a potential choice for further investigation.

#### OX40

OX40 is a TNF family costimulatory that is expressed on activated memory CD4+ T cells and CD4 + FoxP3+ regulatory T cells [71–73] and less expressed on activated CD8+ cells [74]. T cell receptor (TCR) recognition of tumour-specific antigens could induce the upregulation of OX40 expression, and reactivation of primed effector T cells could briefly upregulate OX40 expression again [75, 76]. The stimulation of the OX40/OX40L pathway enhanced the function of effector T cells to kill tumours [77]. Two basic studies explored the importance of timing for optimizing the antitumour effect of PD-1 blockades combined with an agonist anti-OX40 antibody [78, 79]. Shrimali et al. demonstrated that the concurrent addition of a PD-1 checkpoint blockade to an anti-OX40 antibody offset the antitumour effect of only the anti-OX40 antibody because of the reduction in antigen-specific CD8+ T cell infiltration into the tumour and apoptosis of CD8+ T cells in both the periphery and tumour [78]. Messenheimer et al. found that a sequential combination of an anti-OX40 antibody followed by a PD-1 checkpoint blockade, instead of concurrent treatment, significantly augmented the therapeutic efficacy, which depended on both CD4+ and CD8+ T cells [79]. Both studies provided important suggestions for the sequence of combination immunotherapy in clinical trials.

#### Radiotherapy (RT)

Concurrent chemoradiotherapy is the standard treatment for GBM patients. In recent years, an in-depth study on the interaction between RT and the tumour immune microenvironment revealed that RT could induce the immunogenic death of tumour cells and reprogram the TME through recruiting and activating effector T cells [80]. Klug et al. demonstrated that low doses of RT (≤ 2 Gy) reprogrammed TAMs to an M1 phenotype and normalized the tumour blood vessels [81]. Some experiments found that RT could attenuate the suppressive phenotype of Tregs. Cao et al. demonstrated that RT could suppress Treg cell proliferation, especially at a dose of 0.94 Gy [82]. Several studies have demonstrated that different RT doses and fractions can be combined with costimulatory or coinhibitory T cell receptors to increase the homing capacity and activity of T cells. Zeng et al. tested the efficacy of the combination treatment of a PD-1 blockade with stereotactic radiosurgery (SRS) in glioblastoma mouse models and found that combination therapy was superior to either of the single treatments in terms of survival improvement through increasing tumour infiltration by cytotoxic T cells and decreasing Treg activity [83]. The antitumour effects of the triple therapy of a TIM-3 blockade with SRS and a PD-1 checkpoint blockade were also explored in glioblastoma mouse models. Kim et al. demonstrated that triple therapy resulted in 100% OS, which was significantly superior to that of dual therapy [84]. Similarly, the process of glioblastoma-infiltrating T cells increasing IDO1 expression may be a potential mechanism that contributed to the PD-1 blockade failure. Ladomersky et al. tested a novel IDO1 enzyme inhibitor with a PD-1 checkpoint blockade and RT and demonstrated that triple therapy cured most glioblastoma in mouse models compared with dual therapy [85]. Therefore, RT-based immunotherapy for glioblastoma patients is worthy of further exploration, especially for patients with recurrent glioblastoma who have limited clinical response to bevacizumab. Nevertheless, RT-based immunotherapy still requires numerous translational research studies before benefitting the survival of glioblastoma patients.

#### Other targets

Type-2 polarized macrophages (M2) are an important pro-tumourigenic phenotype in the TME. Colony-stimulating factor-1 (CSF-1) is responsible for TAM polarization towards the M2 phenotype. Thus, combining inhibitors of CSF-1R with PD-1 blockades may be a potential strategy to overcome the immunosuppressive context [47]. In addition, dendritic cell (DC) vaccines combined with PD-1 checkpoint blockades also achieved OS benefits in glioblastoma mouse models via directly altering tumour-infiltrating myeloid cell (TIM) expression of key chemotactic factors associated with promoting increased TIL infiltration after vaccination [86]. In addition, neoantigens derived from tumour-specific protein-encoding mutations can induce a strong immune response and are unaffected by central tolerance. Keskin et al. demonstrated that a strategy that uses multi-epitope, personalized neoantigen vaccinations is feasible for glioblastoma due to neoantigen-specific CD4+ and CD8+ T cell responses and the increase of TILs [87]. Aurisicchio et al. found that immune checkpoint inhibitors (ICIs) also act by inducing de novo responses against tumour neoantigens [88]. Keskin et al. Thus, the combination of cancer vaccines targeting neoantigens with ICIs is also a worthwhile treatment regimen. In addition, studies have shown that Tregs could inhibit T cell activation and proliferation through downregulating IL-2 production [48]. Furthermore, Tregs also inhibit IFN-γ production and promote T_H_2 cytokine secretion to further maintain the anergic status of T cells and propagate the regulatory phenotype of Tregs [49, 89, 90]. The secretion of CCL2 and CCL22 in glioblastoma tumour cells could facilitate infiltration and recruitment in the TME [34, 91]. Thus, targeting Tregs may be a potential strategy to enhance the efficacy of PD-1/PD-L1 checkpoint blockades in glioblastoma. Similarly, Wang et al. synthesized microenvironment-responsive nanoparticles (P) with IL-12 payload (IL-12 ⊂P1) to release IL-12 and convert the pro-tumourigenic M2 phenotype to the anti-tumourigenic M1 phenotype in the TME [92]. Saha et al. further studied the intratumoural delivery of oncolytic virus expressing IL-12 combined with CTLA-4 and PD-1 dual checkpoint blockades and found that triple therapy cured most gliomas in mouse models [93]. In addition, TGF-β is closely related to malignant biological behaviour and the immunosuppressive microenvironment of glioblastoma [94]. A phase Ib study of an anti-TGF-β antibody in combination with a PD-1 checkpoint blockade in advanced solid tumours including GBM is in progress (NCT02423343).

### Biomarkers

Several clinical studies have confirmed the predictive effect of PD-L1 expression on the response rate of ICIs in patients with NSCLC, melanoma, colorectal cancer, renal-cell carcinoma, and prostate cancer [95]. Although PD-L1 is highly expressed on GBM [13, 14], the predictive effect of PD-L1 expression on the efficacy of ICIs in glioblastoma remains unclear. Furthermore, the prognostic value of PD-L1 expression in glioblastoma on survival outcomes also demonstrated contradictory results [13, 14, 96, 97]. It can be inferred that the PD-1/PD-L1 signalling pathway does not play a critical role in the development and progression of glioblastoma and may be affected by other factors. Therefore, it is difficult to obtain satisfactory results by simply blocking the PD-1/PD-L1 pathway.

The correlation between MMR protein deficiency or POLE mutations and ideal therapeutic efficacy to PD-1 checkpoint blockades in patients with glioblastoma was first reported in two case reports [98, 99]. The ideal therapeutic effect of PD-1 checkpoint blockades benefits from the patients’ high mutation load. Furthermore, as a tumour-specific neoantigen, the EGFRvIII mutation occurs in 31–50% of patients with glioblastoma, and 37–86% of tumour cells express the mutated protein [100–102]. EGFRvIII promotes tumour cell growth and invasion and plays a negative prognostic role in glioblastoma patient survival [103–106]. Considering the high expression rate and oncogenic characteristics of EGFRvIII, it may be an ideal target and biomarker for glioblastoma immunotherapy. In addition to EGFRvIII, IDH1/2 mutations also play an important role in glioblastoma. The incidence of IDH1/2 mutations in primary glioblastoma is approximately 5%, but in recurrent glioblastoma, the incidence is approximately 84.6%. The efficacy of PD-1/PD-L1 checkpoint blockades depends on the effective infiltration of the activated T lymphocytes in tumours. Kohanbash et al. confirmed that the activation mutation of IDH1/2 inhibited the accumulation of effector T cells in glioma tumours and that treatment with IDH1 inhibitors significantly enhanced the infiltration of effector T cells [107]. Thus, the activation mutation of IDH1/2 in glioma provides a new angle to promote the clinical efficacy of PD-1/PD-L1 checkpoint blockades.

In addition to these molecular features, TILs and NK cells are also considered predictors of PD-1/PD-L1 checkpoint blockade immunotherapy efficacy. Although TILs usually represent an exhausted status and show sparse-to-moderate density infiltration in glioblastoma, a certain amount of TILs in the TME is still the basis for the efficacy of checkpoint blockade immunotherapy. Thus, the prognostic and predictive role of TILs requires further exploration. In addition to TILs, NK cells have been shown to play an indispensable role in PD-1/PD-L1 checkpoint blockades. Hsu et al. demonstrated that NK cells were inhibited by PD-1/PD-L1 interactions and recovered with PD-1/PD-L1 checkpoint blockades. This result indicated that NK cells could directly respond to PD-1/PD-L1 checkpoint blockades [108]. In addition to a single molecular marker, Cheng et al. also analysed genetic data from 297 glioblastoma samples from a bioinformatic perspective. They identified 8 genes (FOXO3, IL6, IL10, ZBTB16, CCL18, AIMP1, FCGR2B, and MMP9) with significant prognostic value in glioblastoma. A local immune-related risk signature was adopted to divide patients into two groups: low-risk patients with high expression levels of protective genes (FOXO3, AIMP1, and ZBTB16) and high-risk patients with high expression levels of risky genes (IL6, IL10, CCL18, FCGR2B, and MMP9) [109]. Thus, it is worth exploring which group of patients is more likely to benefit from PD-1/PD-L1 checkpoint blockades. The available studies presenting candidate biomarkers are summarized in Table 2.

**Table 2:** Candidate biomarkers for checkpoint blockade immunotherapy in glioblastoma

Biomarkers	N	Population	Express positivity on tumor cells	Results	Ref
PD-L1	135	Newly diagnosed glioblastoma (*N* = 117)	88.0%	No association between PD-L1 positivity and OS	[5]
		Recurrent glioblastoma (*N* = 18)	72.2%		
PD-L1	94	glioblastoma	61.0%	PD-L1 positivity associated with poor OS	[6]
PD-L1	54	glioblastoma	31.5%	PD-L1 positivity associated with worse OS	[77]
TILs	135	glioblastoma	Sparse-to-moderate in 72.6%	No association between TILs and OS	[5]
MMR deficiency	2	Recurrent glioblastoma	high neoantigen loads (> 20,000 mutations)	Nivolumab monoclonal antibody has significant clinical response	[78]
POLE deficiency	1	glioblastoma	high neoantigen loads	Pembrolizumab monoclonal antibody has objective radiographic response and lymphocyte infiltration	[79]
EGFRvIII	196	glioblastoma	31%	In subset of OS≥1 year, EGFRvIII positivity associated with poor OS	[86]

### Challenges of PD-1/PD-L1 checkpoint blockades in glioblastoma

PD-1/PD-L1 checkpoint blockades are gradually becoming an effective therapeutic strategy for several types of tumours. Whereas its therapeutic efficacy in glioblastoma remains to be elucidated, several preclinical studies have demonstrated optimal outcomes. The main challenges are as follows: First, there are discrepancies between different genomic subtypes or molecular profiles in the response to PD-1/PD-L1 checkpoint blockades. An analysis of PD-L1 expression in glioblastoma samples has revealed that the MES subtype of glioblastoma has higher PD-L1 expression [13]. In addition to PD-L1, the MES subtype displayed an immunogenic status with gene mutations along with a high neoantigen burden, which increased the response to PD-1/PD-L1 checkpoint blockades. Thus, a computational characterization of the ability of each subtype to respond to PD-1/PD-L1 checkpoint blockades is urgently needed.

Second, there is a challenge in the selection of PD-1/PD-L1 checkpoint blockades for primary versus recurrent glioblastoma. The phase III clinical trial Checkmate 143 reported that PD-1 monoclonal antibody (nivolumab) monotherapy does not significantly improve overall survival time compared with bavacizumab in recurrent glioblastoma patients who were previously treated with chemotherapy and radiotherapy [26]. A study including 22 patients with recurrent glioblastoma also demonstrated no clinical response to pembrolizumab, which targets PD-1 [110]. Then, the efficacy of nivolumab, which targets PD-1, was explored in newly diagnosed glioblastoma patients. Lim et al. [111] assessed the safety and tolerability of nivolumab in combination with RT ± temozolomide (TMZ) in two cohorts. The cohort with TMZ (1c) enrolled 55 patients, including 12 patients with methylated MGMT and 43 patients with unmethylated MGMT. The cohort without TMZ (1d) enrolled 58 patients with unmethylated MGMT. The discontinuation of treatment in the 1c and 1d groups was mostly due to radiographic progression (1c: 50% in the methylated subgroup, 37% in the unmethylated subgroup; 1d: 64%), study drug toxicity (8, 9%; 10%), or patient decision (8, 14%; 0%). The most common (≥30% of patients) AEs were headaches (42, 47%; 41%) and seizures (25, 16%; 31%), which demonstrated that nivolumab was well-tolerated in newly diagnosed glioblastoma patients, and the rate of AEs were consistent with the neurological AE frequency in other reports. Additionally, no drug toxicity-induced deaths were reported. However, the survival data need to be followed up further. All of these data support the continued exploration of nivolumab + RT ± TMZ in newly diagnosed glioblastoma patients in ongoing clinical trials, including Checkmate 498 (NCT02617589) and Checkmate 548 (NCT02667587). From Checkmate 143, we found discrepancies in tolerability and drug toxicity between newly diagnosed glioblastoma patients and recurrent glioblastoma patients. Thus, the clinical outcomes are also worth looking into.

Third, the identification of the optimal combination and sequence for combination therapy is challenging work. Although several preclinical studies have achieved optimal ORR in glioblastoma mouse models with antibodies targeting PD-1/PD-L1, CTLA-4, TIM-3 LAG-3, IDO, or OX-40 [20, 34, 74, 77], there is still a long time period before these strategies are approved for clinical use. In addition, the optimal combination strategy and the sequence of combination therapy for primary glioblastoma versus recurrent glioblastoma also needs to be identified. Considering that different antibodies, which targeted PD-1, CTLA-4, LAG3, etc., and different vaccines triggered different alterations in immune cells and the secretion of key chemotactic factors in the TME, the optimal combination strategy should be able to synergize with PD-1/PD-L1 checkpoint blockades to induce tumour cell immunogenicity and stimulate effective antitumour responses. Furthermore, if clinical trials confirm discrepancies in response rates for PD-1/PD-L1 checkpoint blockades between primary and recurrent glioblastoma tumours, a panel describing the landscape of molecular characteristics of the TME in both types of glioblastoma patients is worth investigating.

## Conclusion

PD-1/PD-L1 checkpoint blockades have exhibited significant efficacy in several types of tumours [112–116]. Nevertheless, current clinical data demonstrated that the clinical efficacy of PD-1/PD-L1 checkpoint blockades in glioblastoma is not significant. Many clinical trials are ongoing to evaluate the safety, tolerability and efficacy of PD-1/PD-L1 checkpoint blockades combined with antibodies targeting CTLA-4, TIM-3 LAG-3, IDO, or OX-40, vaccines, and RT. However, many factors need to be taken into consideration. First, due to the negative regulation of immunosuppressive factors, glioblastoma tumours are called cold tumours and have a low immunogenic nature. Thus, the combination of nivolumab with bevacizumab did not show better efficacy over bevacizumab alone in recurrent glioblastoma patients. Second, the optimal combination strategy and the sequence of combination therapy for primary glioblastoma versus recurrent glioblastoma also needs to be identified. Third, treatment-related AEs cannot be ignored. From Checkmate 143, we found that nivolumab in combination with CTLA-4 monoclonal antibody (ipilimumab) resulted in 40% of recurrent glioblastoma patients having intolerable treatment-related severe side effects. However, nivolumab in combination with RT ± TMZ was well-tolerated in newly diagnosed glioblastoma patients. Thus, the checkpoint blockade-related adverse effects, including inflammatory and autoimmune events, were major obstacles to achieving optimal efficacy.

Overall, the establishment of a panel describing the landscape of the molecular characteristics of the glioblastoma TME for PD-1/PD-L1 checkpoint blockade-based combination therapies is of the most importance to maximize the survival benefits and move treatment towards precision medicine.

## Abbreviations

CNS: Central nervous system
CTLA-4: Cytotoxic T lymphocyte-associated antigen-4
DC: Dendritic cell
IDO: Indoleamine 2,3 dioxygenase
IL-10: Interleukin-10
LAG-3: Lymphocyte activation gene-3
M2: Type-2 polarized macrophages
MDSC: Myeloid-derived suppressor cell
PD-1: Programmed death-1
PD-L1: Programmed death-ligand 1
POLE: Polymerase epsilon
TAM: Tumor-associated macrophage
TGF-β: Transforming growth factor-β
TILs: Tumor infiltrating lymphocytes
TIM: Tumor-infiltrating myeloid cell
TIM-3: T cell immunoglobulin domain and mucin domain-3
TML: Tumor mutational load
TMZ: Temozolomide
VEGF: Vascular endothelial growth factor
